# Sustainable Medical Materials: AI-Driven Assessment for Mechanical Performance of UVC-Treated Date Palm Epoxy Composites

**DOI:** 10.3390/polym17081125

**Published:** 2025-04-21

**Authors:** Mohamed A. Aboamer, Abdulrahman Hakami, Meshari Algethami, Ibrahim M. Alarifi, Tarek M. A. A. El-Bagory, Ahmad Alassaf, Bakheet A. Alresheedi, Ahmad K. AlOmari, Abdulaziz Abdullah Almazrua, Nader A. Rahman Mohamed

**Affiliations:** 1 Department of Medical Equipment Technology, College of Applied Medical Sciences, Majmaah University, Majmaah 11952, Saudi Arabia; m.aboamer@mu.edu.sa (M.A.A.);; 2 Medical Planning of Ministry of Health, Riyadh 11176, Saudi Arabia; 3 Health Services of the Ministry of Defense, Riyadh 56688, Saudi Arabia; 4 Department of Mechanical and Industrial Engineering, College of Engineering, Majmaah University, Majmaah 11952, Saudi Arabia; 5 Department of Mechanical Design, Faculty of Engineering Mataria, Helwan University, El-Mataria 11724, Egypt; 6 Biomedical Technology Department, College of Applied Medical Sciences in Al-Kharj, Prince Sattam Bin Abdulaziz University, Al-Kharj 11942, Saudi Arabia; a.alomari@psau.edu.sa; 7 Administrative and Operational Services Department, Al Rass General Hospital, Al Rass 58883, Saudi Arabia; 8 Biomedical Engineering Department, Faculty of Engineering, Misr University for Science and Technology (MUST), Giza 12568, Egypt

**Keywords:** AI-assisted analysis, recycled composites, UVC disinfection, mechanical properties, medical applications

## Abstract

This study investigates the AI-assisted analyses of radiation disinfection effects on the mechanical properties of recycled date kernel powder–epoxy composites for medical applications, utilizing Euclidean distances and the k-nearest neighbor (KNN) algorithm. Tensile and compression tests were conducted on twenty specimens following ASTM standards, with the data analyzed using a *t*-test to evaluate the impact of the UVC disinfection process on the material’s mechanical properties. The application of AI through the KNN algorithm successfully identified the three most representative curves out of five for both tensile and compression tests. This targeted curve selection minimized variability and focused on the most relevant data, enhancing the reliability of the analysis. Following the application of UVC and AI, tensile tests showed a 20–30% increase in ultimate stress. Similarly, compression tests revealed a 25% increase in transition stress, an 18–22% improvement in ultimate stress, and approximately a 12% rise in fracture stress. This research underscores the potential of combining AI, sustainable materials, and UVC technology to develop advanced composites for medical applications. The proposed methodology offers a robust framework for evaluating material performance while promoting the creation of eco-friendly, high-performance materials that meet the stringent standards of medical use.

## 1. Introduction

The date palm (*Phoenix dactylifera*) is a valuable crop predominantly cultivated in Middle Eastern countries such as Saudi Arabia, Iraq, and Iran, as well as in regions of the United States like California. This palm species holds significant economic and environmental importance. Date palms are primarily grown for their fruit, which is consumed in various forms, including fresh, dried, and processed forms. Globally, approximately 100 million date palm trees exist, with around 62 million concentrated in the Middle East and North Africa [1]. The cultivation of date palms generates substantial agricultural waste, particularly in the form of date palm kernels, with thousands of tons produced annually [1].

Agricultural residues, such as wheat straw, have been utilized for various applications over the years [2,3,4]. Similarly, the abundant residues from date palms, common in the Persian Gulf region (including Iran and Saudi Arabia) and Iraq, represent an alternative source of organic material [5,6]. However, a significant portion of this waste is often left in agricultural areas or burned, leading to environmental and health concerns [7,8]. Utilizing these natural resources in bio-applications presents an opportunity to meet the growing demand for recyclable and biodegradable materials. Moreover, date palm residues hold potential for applications beyond their current use, including energy generation [9,10].

### 1.1. Background

#### 1.1.1. Composite Natural Materials

Natural composite materials have attracted significant attention for medical applications due to their unique properties, such as biocompatibility, biodegradability, and the potential to reduce the environmental footprint of medical devices and implants. These materials typically consist of natural fibers or particles combined with a matrix, often a biodegradable polymer, forming composites suitable for diverse medical uses [11].

Examples of the use of natural composite materials in medical applications include the following:

- Biodegradable Surgical Sutures: Natural composite materials are employed in the production of biodegradable surgical sutures. Polylactic acid (PLA), a biodegradable polymer matrix, is frequently reinforced with natural fibers like flax or silk to enhance mechanical properties [11].
- Wound Dressings: In wound dressings and bandages, natural fiber composites such as cotton or cellulose are integrated into biopolymer matrices for their biocompatibility and healing properties [11].
- Bone Implants and Scaffolds: Composite materials reinforced with natural fibers, such as bamboo, jute, or sisal, combined with biodegradable polymers, mimic the mechanical properties of bone and support tissue regeneration [11].
- Other Medical Applications: Natural composite materials are also used in dental materials, drug delivery systems, orthopedic devices, prosthetics, tissue engineering scaffolds, and cardiovascular devices. These materials provide an optimal balance of strength, flexibility, and biodegradability, making them highly promising for sustainable medical practices [11].

#### 1.1.2. UV Irradiation and Its Effects on Mechanical Properties

UV irradiation, commonly known as UV light, is a form of electromagnetic radiation with wavelengths shorter than visible light but longer than X-rays. Among its various types, UVC radiation, with wavelengths ranging from 100 to 280 nm, is the most effective for killing germs, making it particularly valuable for medical disinfection [12,13,14,15,16].

The effects of UV irradiation on the mechanical properties of biomaterials, including those derived from date palm residues, have become an area of increasing interest. Since UVC is widely used for sterilization, it is crucial to understand its impact on material properties to ensure the structural integrity and functionality of medical devices and implants following disinfection.

### 1.2. Literature Review

#### 1.2.1. Date Palm Seeds in Industrial Applications

Date palm seeds are rich in dietary fiber, carbohydrates, phenols, proteins, and essential minerals such as magnesium, potassium, and calcium [17]. These compounds offer numerous biological benefits, including antibacterial, antioxidant, and antiviral properties [18,19]. The industrial sector has increasingly embraced organic materials, particularly natural fillers and composites, due to their sustainability, cost-effectiveness, and excellent mechanical properties [20,21,22].

Extensive research has explored the use of natural fillers, such as olive husks, nutshells, talc, and tamarind kernel powder, in composite materials. These studies have demonstrated that natural fillers not only enhance mechanical properties but also reduce production costs [23,24,25,26,27,28]. For example, hybrid polypropylene (PP) composites reinforced with graphite and clay exhibit significantly improved mechanical performance compared to the polymer matrix alone [29,30]. Similarly, research on using coffee husks as fillers for thermoplastic materials has shown enhancements in both mechanical and thermal properties [31,32].

Dumitru Bolcu et al. studied the mechanical behavior of composite materials with a hybrid epoxy matrix reinforced by dammar and chopped wheat straw. Mechanical parameters such as the modulus of elasticity, tensile strength, and elongation at fracture were evaluated using tensile and compression tests. Water absorption and loss were also examined. These composites exhibited limited flexural strength but showed potential for applications in construction (e.g., paneling) and furniture [33].

Aboamer et al. reported four investigations on the effect of UV irradiation on the mechanical and chemical properties of composite materials. The first study focused on the impact of UVC disinfection on composite polymer magnets. Tensile and compression tests conducted per ASTM standards revealed increased tensile strength (0.41 kN to 0.58 kN) and compression yield force (4.9 kN to 6 kN). EDS analyses showed an increase in carbon content (71.69% to 78.56%), correlating with improved material hardness [34].

The second study evaluated PLA materials under UVC (254 nm) and combined UVC/UVB (310 nm) disinfection. Significant differences in tensile yield displacement (*p* = 0) and fracture displacement (*p* = 0.047) were observed, as well as in compression yield displacement (*p* = 0.04). These findings highlight the potential of hybrid UVC/UVB disinfection for medical applications [35].

The third study examined the effects of UVC radiation on ABS materials using tensile, compression, and bending tests. The ANOVA test showed significant differences in tensile strength (*p* = 0.012), with average ultimate stresses of 34.5 MPa (tensile), 25.4 MPa (compression), and 24.5 MPa (bending) [14].

The fourth study assessed the mechanical properties of LDPE and HDPE before and after UVB irradiation. HDPE showed greater impacts than LDPE, with significant differences in yield stress (*p* = 3.008 × 10^−4^), ultimate stress (*p* = 2.5 × 10^−4^), and break stress (*p* = 0.0075) [36].

#### 1.2.2. AI Applications in Biomaterials

Artificial intelligence (AI) has emerged as a transformative tool in biomaterial research, with recent studies showcasing its applications. One notable study highlights the growing importance of machine learning (ML) and deep learning (DL) in predicting material properties and designing novel materials [37,38]. As the complexity of modern materials increases, traditional methods often struggle to explore the vast design spaces required to optimize mechanical behavior. In contrast, ML models trained on large datasets that connect structure, properties, and function offer a faster and more efficient approach to material design.

Another study underscores the role of AI in enhancing the accuracy of mechanical property assessments, particularly through unsupervised learning techniques that identify and exclude outliers. By focusing on the most reliable data, AI ensures robust evaluations of material suitability, a critical requirement in biomedical applications where precision is paramount [37].

For the first time, AI has been employed to reduce the number of tensile and compression test samples by detecting and excluding unreliable curves that could introduce deviations. This innovative approach improves the accuracy of mechanical testing while minimizing errors, thereby providing dependable data. This advancement sets a new standard for material testing in biomedical applications, demonstrating the potential of AI to revolutionize the field.

### 1.3. Problem Statement

Can the combination of recycled date kernel powder and epoxy produce a durable material suitable for medical applications, maintaining its effectiveness after UVC disinfection? Furthermore, how can artificial intelligence (AI) enhance the accuracy of analysis by identifying and excluding unreliable data?

### 1.4. Objectives of the Study

The objectives of this study are as follows:

1. To explore and repurpose locally available materials, specifically recycled date kernel powder, from Middle Eastern countries for potential use in medical applications.
2. To design and conduct tensile and compression tests to evaluate the impact of UVC radiation disinfection on the mechanical properties of these materials, following ASTM standards.
3. To assess the suitability of these materials for prolonged medical use by measuring changes in their mechanical properties post-disinfection and evaluating their durability over time.
4. To apply AI-assisted analysis techniques to enhance the accuracy and reliability of mechanical property assessments by filtering out inconsistent test results.

## 2. Materials and Methods

**Figure 1:**
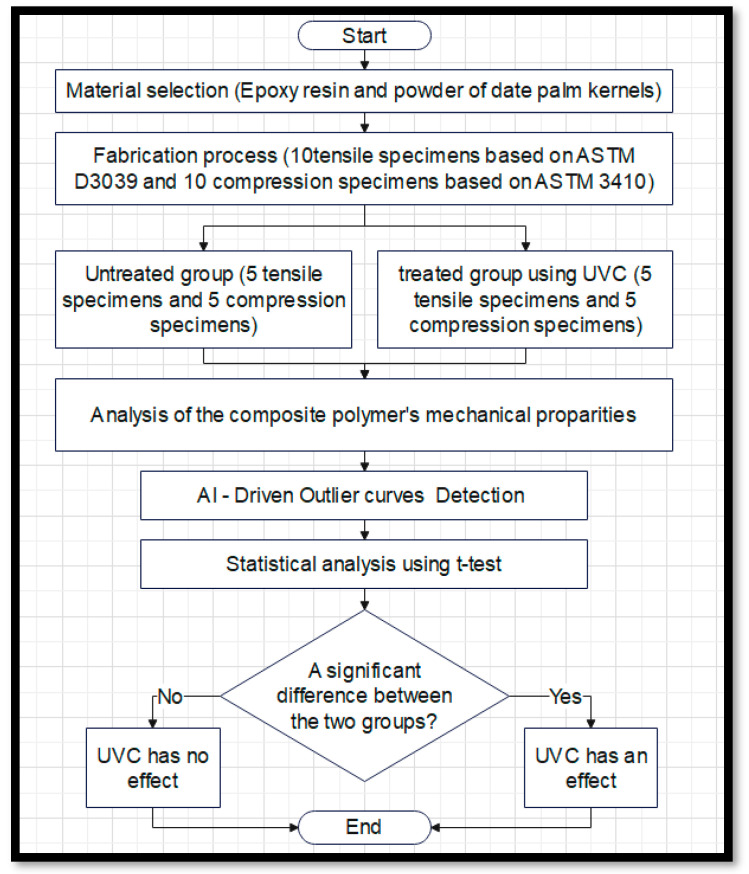
Block diagram of the proposed system.

The flowchart outlines a comprehensive process for evaluating the effect of UVC irradiation on the mechanical properties of a composite material made from epoxy resin and date palm kernel powder. Below is a detailed explanation of each step, as shown in Figure 1:

Start: The process begins with the initiation of the experimental procedure to understand how UVC disinfection impacts the composite material’s mechanical properties.

Material selection (epoxy resin and date palm kernel powder): In this step, the materials to be studied are selected. The chosen materials include a combination of epoxy resin and powdered date palm kernels selected for their potential to create sustainable composites with applications in medical fields.

Fabrication process (10 tensile specimens based on ASTM D3039 and 10 compression specimens based on ASTM D3410): The materials are fabricated into test specimens. Specifically, 10 tensile specimens are prepared according to ASTM D3039 (Standard Test Method for Tensile Properties of Polymer Matrix Composite Materials), and 10 compression specimens are prepared following ASTM D3410 (Test Method for Compressive Properties of Polymer Matrix Composite Materials). These standards ensure consistent and reliable testing [39,40].

Untreated group (5 tensile specimens and 5 compression specimens): The specimens are divided into two groups. The untreated group consists of 5 tensile and 5 compression specimens that are not subjected to UVC irradiation, serving as the control group in the experiment.

Treated group using UVC (5 tensile specimens and 5 compression specimens): The second group undergoes UVC irradiation. Like the untreated group, it contains 5 tensile and 5 compression specimens. This group allows researchers to assess the impact of UVC irradiation on the material’s properties.

Analysis of the composite polymer’s mechanical properties: In this step, both groups (untreated and treated) are analyzed for their mechanical properties, typically involving the measurement of parameters such as tensile strength, compressive strength, modulus of elasticity, and other relevant mechanical factors. The results from both groups are then compared.

AI-driven outlier curve detection: To enhance the accuracy of the analysis, AI is used to detect and exclude outlier data that could distort the results. Outliers might arise from manufacturing defects, measurement errors, or inconsistencies. By using AI-driven techniques, only the most reliable data are used for further statistical analysis, improving the overall quality of the study.

Statistical analysis using *t*-test: After removing outliers, a *t*-test is performed. This statistical method compares the means of the two groups (untreated and treated) to determine whether there is a statistically significant difference between them. The *t*-test helps confirm whether UVC treatment has a meaningful impact on the mechanical properties of the material.

Is there a significant difference between the two groups? This decision point checks whether the *t*-test results show a statistically significant difference between the untreated and treated groups.

Yes: If a significant difference is found, the conclusion is that UVC irradiation affects the mechanical properties of the composite material.

No: If no significant difference is found, the conclusion is that UVC irradiation does not impact the material’s mechanical properties.

End: The process concludes based on the statistical analysis, leading to a final determination regarding the influence of UVC treatment on the material.

### 2.1. Material Selection and Specimen Fabrication

#### Molding Process

**Figure 2:**
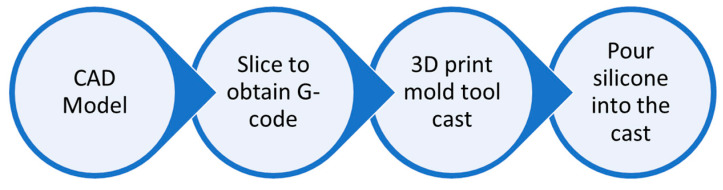
The process flow of the silicone mold tool’s fabrication.

The procedures involved in fabricating the silicone mold closely resemble those outlined in a prior study [34]. The silicone materials for this experiment, which have a 45 min to 1 h working time and a 24 h curing time, were obtained from Casting Craft. Figure 2 illustrates the flow diagram of the mold-making process. To begin the process of creating a silicone mold tool, the first step was to design the mold cast using computer-aided design (CAD) software (SOLIDWORKS Premium 2020 SP0.0) with accurate measurements, and the ASTM D3039 model was used for the tensile model and the ASTM D3410 model was used for the compression model with a length of 25 mm. The CAD file was then saved in a file format known as the standard tessellation language (STL), and the STL file was converted into G-code. After the G-code was successfully created, the mold tool cast was 3D-printed. The bulk of the tool castings for the project were created using fused deposition modeling (FDM) on a Creality Ender 3 printer (Shenzhen Creality 3D Technology Co., Shenzhen, China) with PLA as the printing medium. It is crucial to note that the strength of the mold tool cast is unimportant in this scenario because a very low infill (in this experiment, 25%) may be used to save printing material. Because the component’s finish is reflected in the silicone mold tool, it is an important factor that should not be overlooked.

Epoxy resin was chosen as the material because it can be easily shaped, has an acceptable cost, and is widely accessible on the market. Graffiti Resin is an epoxy resin material that does not contain any volatile organic compounds, and it is also known as zero VOC. The mixing and homogenization ratio of the epoxy resin and hardener was 1:1, and standard processes were followed in the creation of epoxy products. Next, powdered date palm kernels were added and mixed at room temperature at a ratio of 50:50 for the mixing process [34]. The outcome of the previous investigation played a role in the decision-making process regarding the appropriate ratio.

### 2.2. Geometrical Data of Tensile and Compression Specimens

#### 2.2.1. Tensile Specimens

**Figure 3:**
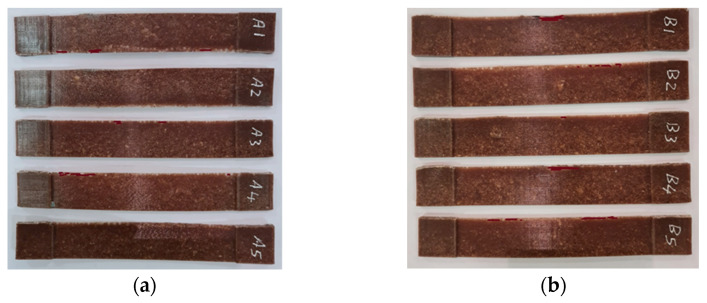
Tensile test specimens (ASTM D3039): (**a**) untreated specimens (A1, A2, A3 A4 and A5) and (**b**) UVC-treated specimens (B1, B2, B3, B4 and B5).

Tensile test specimens were fabricated following the specifications provided in ASTM D3039 under controlled ambient conditions at 25 °C. During the manufacturing process using the mold tool, a total of ten good ASTM D3039 specimens were selected, as shown in Figure 3. Table 1 presents the complete dimensions of ASTM D3039. The specimens were divided into two groups. One group was referred to as the treated group (B1, B2, B3, B4 and B5), whereas the other was denoted as the untreated group (A1, A2, A3, A4 and A5). In Table 1, the first two columns denote the length in millimeters, the third column represents the width in millimeters, the fourth column indicates the thickness in millimeters, and the last column denotes the weight in grams. The study dimensions were selected based on ASTM D3039 standards. The dimensions of the object are as follows: inner length, 125 mm; outer length, 175 mm; width, 25 mm; outer thickness, 5 mm; and inner thickness, 1.5 mm.

#### 2.2.2. Compression Specimens

**Figure 4:**
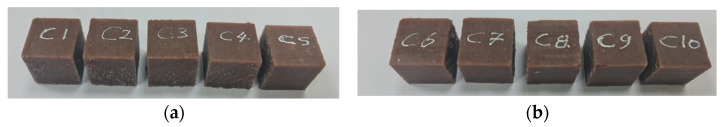
Compression test specimens (ASTM D3410): (**a**) untreated specimens (C1, C2, C3, C4 and C5) and (**b**) UVC-treated specimens (C6, C7, C8, C9 and C10).

At 25 °C, compression test specimens were cast in accordance with ASTM D3410 specifications. Figure 4 depicts the selection of ten good ASTM D3410 specimens during manufacturing using the mold tool. The dimensions of the ASTM D3410 specimens are listed in Table 2. The specimens were separated into two groups. The first group was subjected to UVC irradiation (C6, C7, C8, C9 and C10), while the second group was not (C1, C2, C3, C4 and C5). The first column shows the length in millimeters on each side, and the second column shows the weight in grams.

In ASTM, the standard constant speed is 2 mm/min, and a frequency of 20 Hz is used during data collection, whereas the compression test specimen ASTM D3410 has a test speed of 1.5 mm/min.

Tensile tests were also carried out using a UTM equipped with a 5 kN load cell, and the tensile load *P* [kN] and displacement *L* of the upper crossbar in [mm] were recorded and preserved. Based on this information, it was possible to calculate the stresses in MPa and the non-dimensional strains as follows:

(1) $$\delta =\frac{P}{W\cdot t}\;\;\;\;\;\;\;\;\in =\frac{∆L}{L_{0}}$$

where the initial length is *Lo*, the thickness is *t* (mm), the width is *W* (mm), and the force is *P* (kN).

### 2.3. The Role of UV Radiation in the Deactivation of Viruses and Bacteria

UV light is a type of electromagnetic radiation with wavelengths ranging from 100 to 400 nm. According to ISO 21348:2007 [41], UV radiation can be classified into four types: UVA (300–400 nm), UVB (300–280 nm), UVC (200–280 nm), and vacuum UVV (100–200 nm). UVA radiation is responsible for skin tanning and can cause long-term harm. Psoriasis is a skin condition that may benefit from UVB treatment. As UVV light is absorbed by the atmosphere, it is ineffective for characterizing environmental conditions. UVC irradiation is the most efficient technique for killing bacteria and viruses [16,17,18,19,20].

#### 2.3.1. UV Irradiation Enclosure

**Figure 5:**
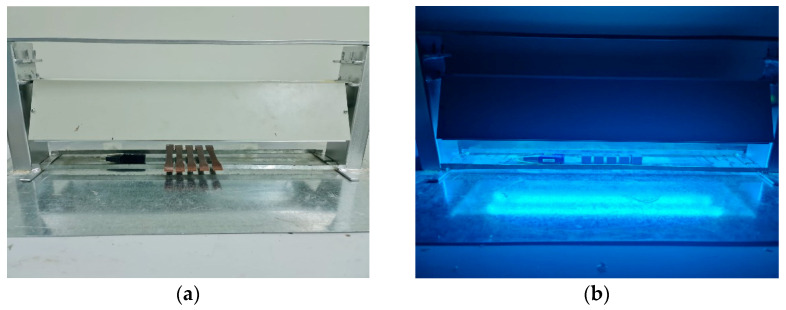
UV irradiation enclosure. (**a**) The adjustable locally made enclosure and the irradiation lamps and (**b**) the arrangement of specimens and the wireless data logger.

A locally constructed irradiating enclosure equipped with two 20 W UV lights with a wavelength of 254 nm was employed for UV radiation exposure. The enclosure can accommodate a variable distance from 8 cm to 16 cm between the surfaces of the two lights, and the surfaces of the measured samples. The adjustable, locally produced enclosure and irradiation lamps with dimensions of 610 × 152 × 108 mm with respect to L × W × H and a weight of 4.5 kg were used [34], as shown in Figure 5. A UV radiometer supplied with ILT equipment was used to monitor irradiance levels across the irradiated zone [34]. Figure 5a and Figure 5b show the placement of the tensile and compression specimens, respectively.

#### 2.3.2. Exposure Time Calculations

The UV-light-emitting instrument had two UVC lamps, each with a power output of 20 W. A distance of 12 cm was measured between the UVC lamps and the surfaces that were being treated. According to a previous study, a dose of 3.7 mJ/cm^2^ at 254 nm is necessary to deactivate the SARS2-COVID virus. A minimum exposure duration of 30 min is required for a 48 W light source, as shown in a recent study [42], to guarantee complete disinfection.

The system’s exposure time in seconds can be calculated using the following equation [43]:

(2) $$Exposure\;Duration\;\left(s\right)=\frac{Dosage\left(\frac{mJ}{{cm}^{2}}\right)}{Lamp\;Power\left(\frac{W}{{cm}^{2}}\right)}=\frac{2\pi LrD}{P}$$

In this context, the variables are defined as follows: *L* represents the length of the UVC lamp, measured in centimeters; *r* denotes the distance, measured in centimeters; *D* represents the UVC dose, measured in millijoules per square centimeter; *P* denotes the power of the emitted UVC light, measured in watts. Appropriate calculations determined that a dose of 3.7 mJ/cm^2^ is necessary to effectively eliminate SARS2-COVID. This dosage was contingent upon the use of UVC lamps with a length of 55 cm, total wattage of 40 W, and a distance of 8 cm between the lamps and the infected surfaces. Based on these calculations, the necessary exposure time was determined to exceed 48 min. Therefore, a duration of 1 h was used as the preferred period for the experiment.

### 2.4. K-Nearest Curve Similarity Estimation for AI-Assisted Pattern Recognition

The process described in this code uses the k-nearest neighbor (KNN) algorithm to find the three most similar curves based on the Euclidean distance. The goal is to determine which curves are closest to each other in terms of their shape or pattern, making it a part of pattern recognition:

1. Reading the data (input curves): The curves represent the different stress–strain relationships of materials (or other mechanical properties):
  - The data for five different curves are read from an Excel file.
  - Each curve has two columns: X-values (strain) and Y-values (stress).
  - Five curves are extracted from the file and stored in a structure that allows for easy comparison later.
2. Combining the curves for distance calculations: All extracted curves are stored in a data structure (such as a cell array) so that they can be processed together:
  - The total number of curves is counted.
  - A distance matrix is initialized to store the pairwise distances between the curves.
3. Calculating pairwise distances using Euclidean distance: The code calculates the Euclidean distance between each pair of curves. The Euclidean distance measures the “closeness” between two sets of points by comparing the difference in Y-values (stress) relative to the same X-values (strain).
  For each pair of curves, the following steps occur:
  - Calculate the difference in Y-values between the two curves at corresponding points.
  - Use the Euclidean distance formula to compute the distance between the two curves.
  The Euclidean distance formula is as follows:
  (3) $$d=\sqrt{\sum_{i=1}^{n}{\left(y_{i}^{1}-y_{i}^{2}\right)}^{2}}$$
  where $y_{i}^{1}$ and $y_{i}^{2}$ are the Y-values of two curves.
  - Store the result in the distance matrix, which keeps track of the distance between every pair of curves.
4. KNN to find the three nearest curves: Once the distance matrix is complete, the k-nearest neighbor (KNN) algorithm is applied to find the three most similar curves:
  - For each curve, sum the distances relative to all other curves. This gives a sense of how close each curve is to the others overall.
  - Sort the curves based on these total distances.
  - Select the three curves with the smallest total distances. These are considered the most similar curves.
5. Visualization of the results: The code then visualizes the results using two main techniques:
  - A heatmap is created to display the distance matrix, where similar curves appear with smaller distances, and dissimilar ones appear with larger distances.
  - The three nearest curves are highlighted both in the heatmap and on a plot that shows all five curves. The three nearest curves are marked with distinct visual indicators (e.g., red lines).
6. Pattern Recognition Aspect: The process of finding the nearest curves based on distance is a form of pattern recognition:
  - The curves represent patterns in how materials behave under stress (i.e., stress–strain relationships).
  - By finding the three most similar curves, the code is identifying materials with similar mechanical properties based on their patterns.

### 2.5. T-Test with Two Samples

A two-sample *t*-test is a statistical hypothesis test used to determine whether there is a significant difference between the means of two independent groups or samples. It is particularly useful for comparing two groups and assessing whether the observed differences in their means are likely due to a real effect or simply the result of random chance. The first component in a two-sample *t*-test is the null hypothesis (H0), which assumes that there is no significant difference between the means of the two groups. This suggests that any observed differences are due to random variations. The second component is the alternative hypothesis (Ha or H1), which should be tested, suggesting that there is a significant difference between the means of the two groups [14,35,36,37]. The following equation was used to compute a two-sample *t*-test:

(4) $$t=\frac{\bar{x}-\bar{y}}{\sqrt{\frac{s_{x}^{2}}{n}-\frac{s_{y}^{2}}{m}}}$$

where *x* and *y* are the sample means; *S_x_* and *S_y_* are their standard deviations; *n* and *m* are the sample sizes [44,45].

## 3. Results and Discussion

### 3.1. Tensile Test for Untreated Specimens

**Figure 6:**
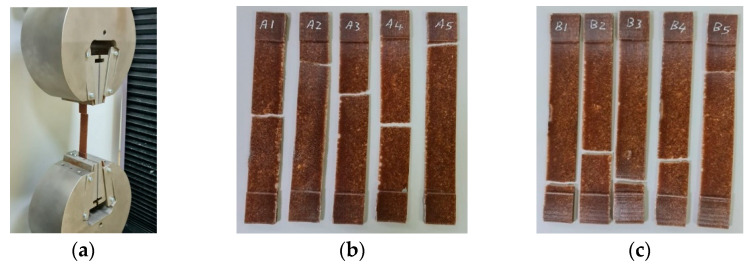
Tensile test setup. (**a**) Universal testing machine (UTM), (**b**) untreated tensile specimens (A1, A2, A3, A4 and A5), and (**c**) UVC-treated tensile specimens (B1, B2, B3, B4 and B5).

The approach specified in ASTM D3039 for testing composite polymers was used in the experiment. Data were collected at a frequency of 20 Hz and a constant speed of 2 mm/min. Figure 6a shows an ASTM D3039 tensile specimen connected to universal testing equipment to determine the mechanical characteristics of the material. Furthermore, the tensile specimens before and after UVC treatment are shown in Figure 6b and Figure 6c, respectively. Figure 6b represents the tensile specimens before UVC treatment (A1, A2, A3, A4 and A5), and Figure 6c represents the tensile specimens after UVC treatment (B1, B2, B3, B4 and B5).

**Figure 7:**
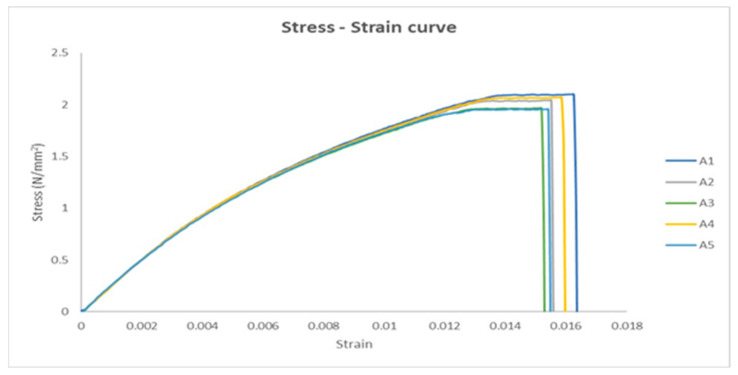
The stress–strain curves for the five untreated UV specimens.

The stress–strain curve is a graphical representation of the mechanical properties of a material, specifically its response to an applied load or stress. Five tensile specimens were investigated as a group without UV irradiation, and the stress–strain curves were obtained to investigate the mechanical properties of the material. The five stress–strain curves are shown in Figure 7. The stress–strain curves have the same shape as the lower slope curve in the ASTM D3039 standard. The stress–strain curve shows the strain along the X-axis and the stress along the Y-axis. Figure 7 shows the most important points, such as the transition point that separates the elastic and plastic regions, the ultimate point that represents the maximum stress axis, and the fracture point that represents the end of the plastic region.

#### After Applying AI-Assisted Curve Selection for the Five Untreated Tensile Curves

**Figure 8:**
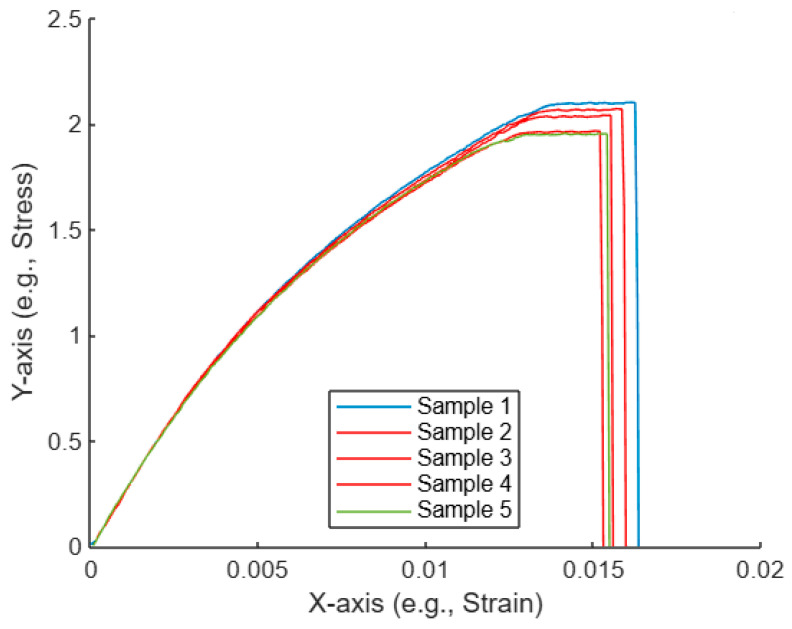
Plot of the five untreated tensile curves, with selected curves highlighted.

Figure 8 shows five curves, each representing a sample with data plotted on the X-axis (strain) and the Y-axis (stress). Some of the curves are highlighted in red, indicating the selected curves that have been determined to be the “nearest” to each other based on the AI-assisted algorithm, which uses Euclidean distances to measure similarity.

1. Curve similarity identification: The AI-assisted algorithm calculates pairwise distances between all curves by measuring the difference in their Y-values (stress) at corresponding X-values (strain). Based on these differences, the algorithm identifies the three curves that are the most similar, or “nearest,” to each other. These selected curves are highlighted in red, while the others are shown in their default colors.
2. Curve behavior: All curves show similar initial trends, where the stress increases with strain, reaching a peak before dropping off. However, slight variations in the peak and the tail of each curve cause differences in their pairwise distances, leading to the distinction between the selected and non-selected curves.
3. AI-assisted decision: The algorithm objectively selects the three nearest curves by minimizing the total distance between the curves in the dataset. These curves are presumably the ones that behave more similarly in terms of stress–strain relationships, indicating they may have undergone similar conditions or material properties during the experiment.
4. Practical implications: By using an AI-assisted approach, the selection of similar curves is automated, which allows for faster, more reliable analysis. In this case, the identified curves could be investigated further to understand what factors caused their similarity. This could be useful in material testing, where identifying the most consistent results is essential for evaluating material properties.

Overall, the AI-assisted curve selection helps streamline the analysis by automatically identifying and highlighting the most comparable curves based on their stress–strain behavior. This is particularly useful for datasets involving multiple samples, where the manual identification of trends could be time-consuming.

**Figure 9:**
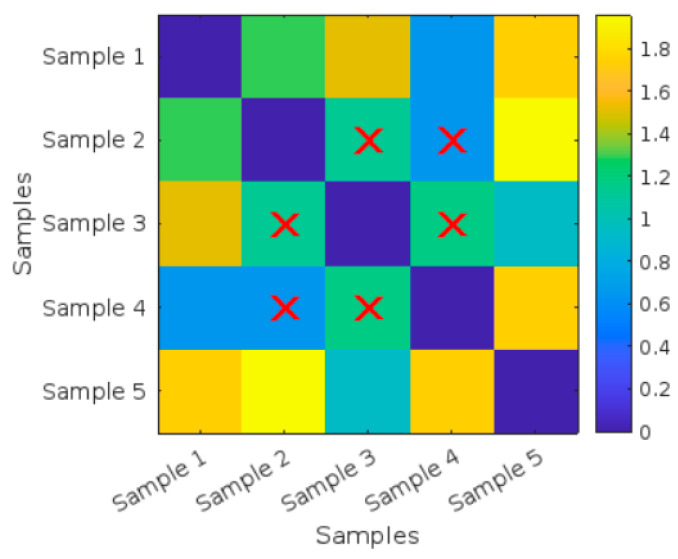
Distance Matrix Heatmap for the five untreated tensile curves.

As shown in the heat map in Figure 9 and the values in Table 3, the image shows a heatmap titled “Distance Matrix Heatmap”, which visualizes the pairwise distances between different samples. Both the X-axis and Y-axis are labeled with “Samples”, indicating the distance relationship between Sample 1 to Sample 5.

A color bar is present on the right side, ranging from 0 to 1.8. Blue represents low values (closer to 0). Yellow represents high values (closer to 1.8). The colors between blue and yellow represent different levels of distances between the samples. The intensity of the colors in the heatmap indicates the magnitude of the distance between each pair of samples.

The diagonal elements (Sample 1 vs. Sample 2, Sample 3 vs. Sample 4, etc.) are represented by dark blue squares, indicating a distance of 0 since each sample is compared to itself. The Distance Matrix Heatmap visualizes the pairwise distances between five different samples: Sample 2, Sample 3, and Sample 4 are identified as having the most similarity (small distances), as highlighted by the red X marks.

In general, both curves provide complementary information regarding the mechanical properties of materials and are often used together to gain a more comprehensive understanding of their behavior under different loading conditions. Stress–strain curves provide researchers with numerous mechanical parameters for the proposed material. Table 4 shows how the tensile specimens were labeled with the letter A, where A1 represents the first specimen and A5 represents the last specimen. This study investigated six mechanical parameters: transition stress (N/mm^2^), transition strain, ultimate strain, ultimate stress (N/mm^2^), fracture strain, and fracture stress (N/mm^2^). The transition stress, transition strain, ultimate strain, ultimate stress, fracture strain, and fracture stress had average values of 1.38 N/mm^2^, 0.01, 0.01, 2.03 N/mm^2^, 0.02, and 1.21 N/mm^2^, respectively.

Note: For sample A2, the ultimate stress and fracture stress are equal, indicating that failure occurred at the maximum load without post-peak deformation.

### 3.2. Tensile Test for UV-Treated Specimens

**Figure 10:**
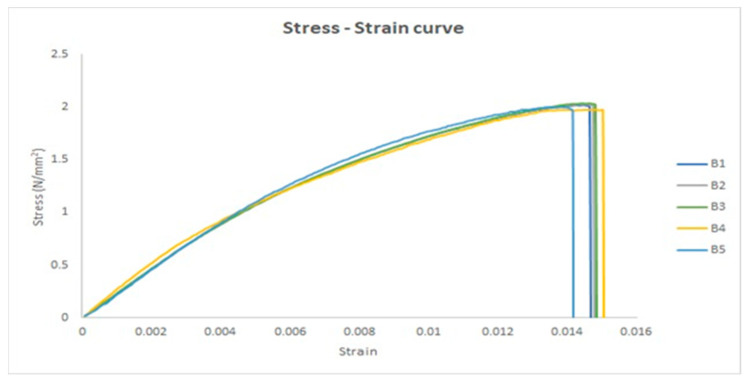
The stress–strain curves for the five UV-treated specimens.

After subjecting the five tensile test specimens to UV irradiation or after disinfecting them with UVC irradiation at 254 nm for 60 min, the results were recorded. In Figure 10, the stress–strain curves of the five tensile specimens after being treated with UVC for 60 min are presented.

#### After Appling AI-Assisted Curve Selection for the Five UV-Treated Tensile Curves

Behavior of the curves: They start at the origin (0,0), suggesting no strain and no stress initially. The curves increase steadily as strain increases, showing a rise in stress. At approximately x = 0.012 to 0.014, the curves seem to reach a peak and then drop sharply, indicating a point of failure or maximum stress point followed by a reduction.

Selected curves: Sample 1, Sample 2, and Sample 3 are highlighted and drawn in red with a thicker line width. This suggests that these three samples are of particular importance, potentially indicating that they were identified as the most similar or closest to each other based on the analysis (likely using the KNN or Euclidean distance approach mentioned earlier).

**Figure 11:**
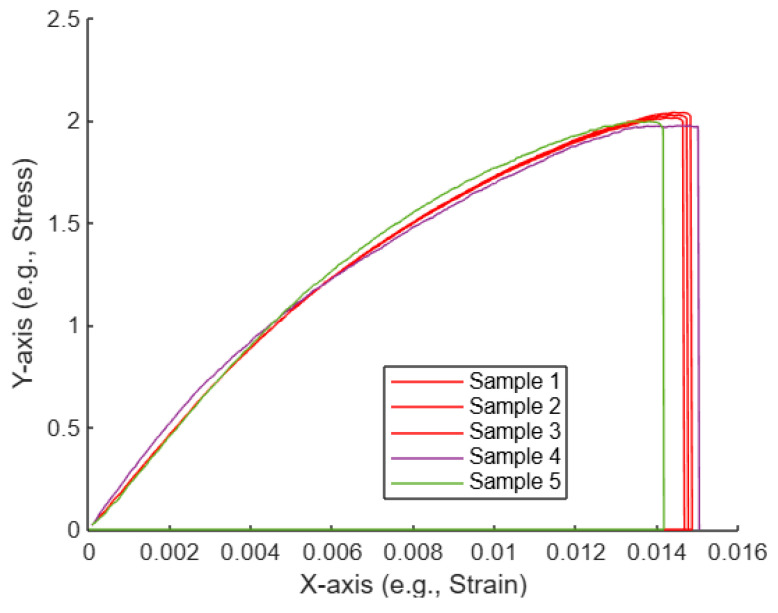
Plot of the five UV-treated tensile curves, with selected curves highlighted.

General observations: The plot shows the tensile behavior of the samples, with a noticeable similarity in their initial behavior but slight variations toward the peak. The selection of three curves that are highlighted implies that these samples have certain similarities that were identified through the analysis. The thick, red lines indicate that these curves were of particular interest, as shown in Figure 11.

**Figure 12:**
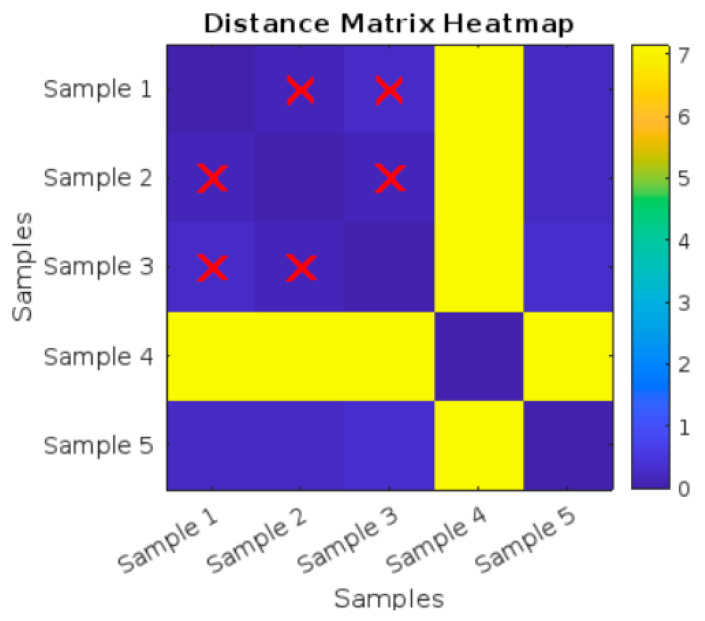
Distance Matrix Heatmap for the five UV-treated tensile curves.

As shown in Figure 12, there are red X marks on certain cells in the heatmap, specifically for Sample 1, Sample 2, and Sample 3. These marks are used to highlight specific distances, likely representing the three nearest neighbors or closest relationships between the samples based on a similarity criterion. The red X marks are placed symmetrically, showing that Sample 1, Sample 2, and Sample 3 have relatively close relationships with each other.

Sample 4 stands out as having high distances (shown in yellow) compared to most other samples, meaning it is significantly different from the other samples. Sample 1, Sample 2, and Sample 3 have smaller distances between each other, as indicated by darker shades and highlighted by the red X marks. The pairwise distances values of the distance matrix heatmap are shown in Table 5.

As presented in Table 6, the mechanical properties of the UV-treated tensile specimens are summarized. The transition stress shows some variability among the specimens, with B 5 having the highest value (1.60 N/mm^2^) and B 4 the lowest (1.38 N/mm^2^). The transition strain and ultimate strain are consistent across all specimens, each having values close to 0.01.

Ultimate stress is slightly higher for B 3 (2.04 N/mm^2^) compared to the other specimens, which have values around 2.00 N/mm^2^. The fracture strain for B 4 is 0.02, which is higher than the others, indicating that B 4 can withstand a greater amount of strain before fracturing. Fracture stress ranges from 1.96 to 2.02 N/mm^2^, showing slight variability between the specimens.

### 3.3. T-Test for Tensile Test Specimens

#### 3.3.1. Statistical Comparison of Mechanical Properties Before AI Analysis

**Figure 13:**
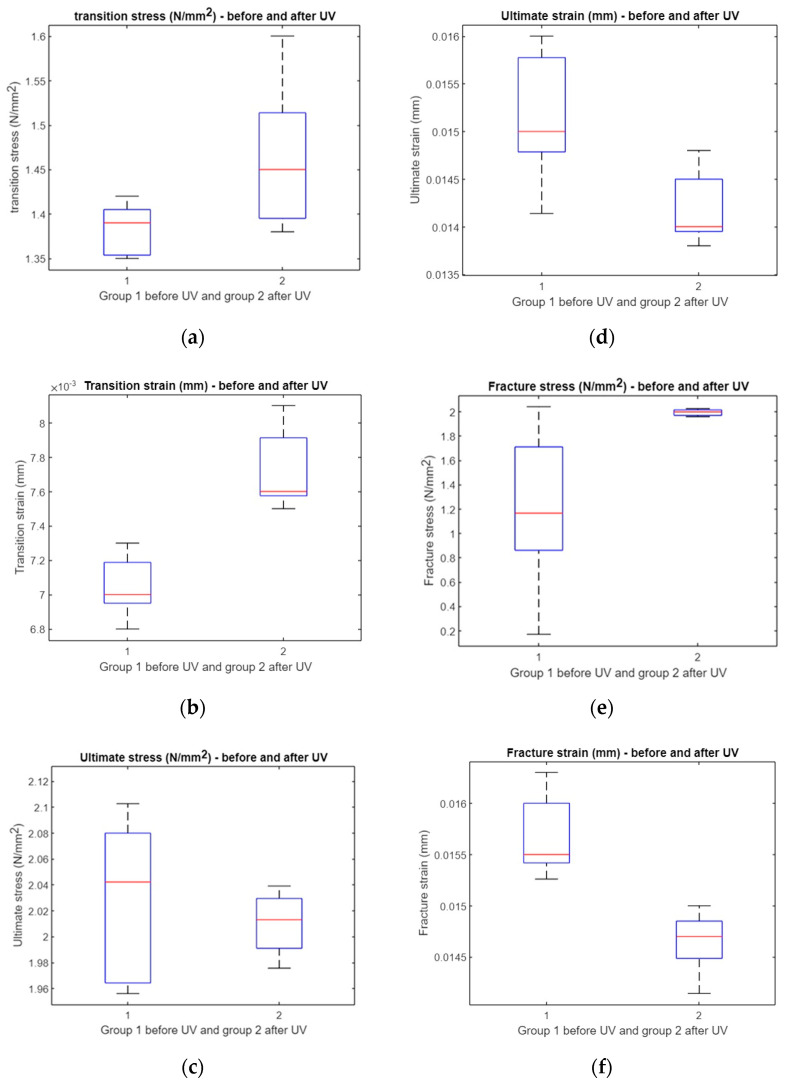
Comparison between UV-treated and untreated samples. (**a**) Boxplot of transition stress before and after UV, (**b**) boxplot of transition strain before and after UV, (**c**) boxplot of ultimate stress before and after UV, (**d**) boxplot of ultimate strain before and after UV, (**e**) boxplot of fracture stress before and after UV, and (**f**) boxplot of fracture strain before and after UV.

A *t*-test was performed to evaluate the impact of UV irradiation on various mechanical properties. The results showed no significant difference in transition stress and ultimate stress before and after UV exposure (*p* = 0.0878 and *p* = 0.0577, respectively). However, significant differences were observed in the transition strain (*p* = 0.0011), ultimate strain (*p* = 0.0306), fracture stress (*p* = 0.0369), and fracture strain (*p* = 0.0022). These outcomes indicate that UV treatment alters certain strain-related parameters more noticeably than stress-related ones (Figure 13). The horizontal red line in the box plot indicates the mean and the box indicates the upper and lower quartiles, with the horizontal black lines representing the minimum and maximum values.

#### 3.3.2. Statistical Comparison of Mechanical Properties After AI Analysis

**Figure 14:**
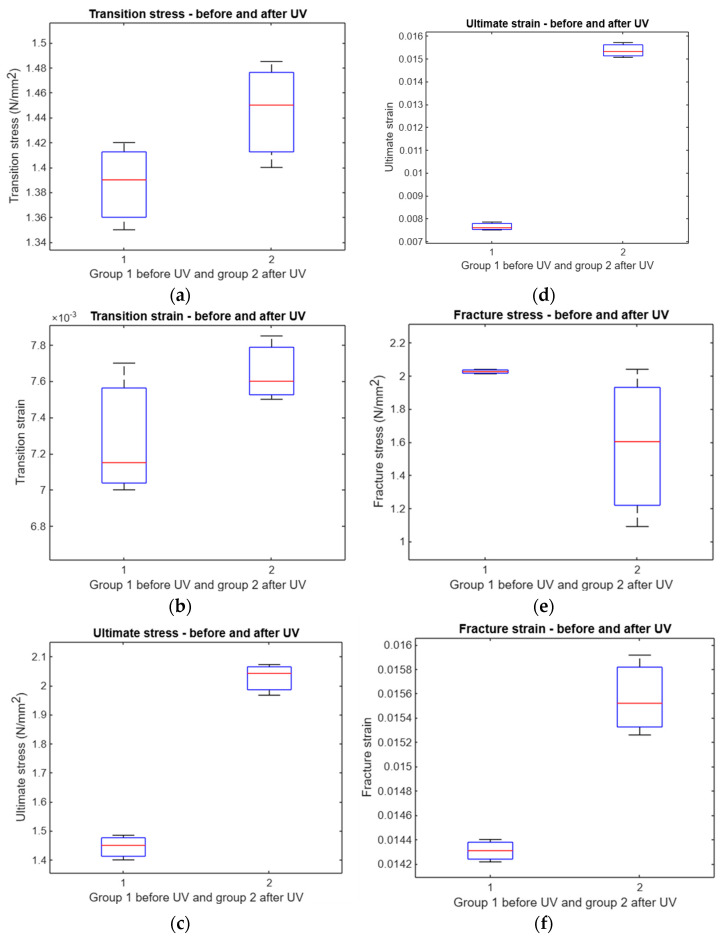
Comparison between treated and untreated samples: (**a**) boxplot of transition stress before and after UV, (**b**) boxplot of transition strain before and after UV, (**c**) boxplot of ultimate stress before and after UV, (**d**) boxplot of ultimate strain before and after UV, (**e**) boxplot of fracture stress before and after UV, and (**f**) boxplot of fracture strain before and after UV.

Following AI-based analysis, most stress and strain measures showed varied responses to UV irradiation. No significant differences were found in transition stress, transition strain, or fracture stress (*p* = 0.1417, 0.1966, and 0.1777, respectively). However, significant differences emerged in ultimate stress and ultimate strain (both *p* = 0), as well as in fracture strain (*p* = 0.0032), suggesting that AI-assisted evaluation enhances detection sensitivities for certain UV-induced mechanical changes (Figure 14).

#### 3.3.3. Comparison Between Before and After AI for Tensile Test Specimens

Here is a comparison table (Table 7) that summarizes the differences before and after AI in terms of statistical significance for each mechanical property:

For transition stress, there is no significant difference both before and after applying AI. For transition strains, it was significant before AI but not significant after AI. Ultimate stress was not significant before AI, but after applying AI, a significant difference was observed. The ultimate strain showed a significant difference both before and after AI. Fracture stress had a significant difference before AI, but no significant difference after AI. Fracture strain remained significant in both conditions.

### 3.4. Compression Test for Untreated Specimens with UV

**Figure 15:**
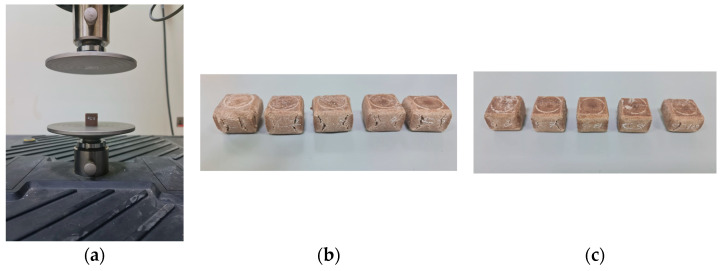
Compression test specimens: (**a**) compression test attached to the compression test machine, (**b**) compression test specimens before UVC treatment, and (**c**) compression test specimens after UVC treatment.

Compression test machines are important tools for quality control and material testing in the construction industry because they provide accurate and reliable data on the strength and durability of building materials. Figure 15 shows how the compression test machine tested the specimen, and Figure 15b, and Figure 15c illustrate the shape of the compression specimens after the compression test was finished.

**Figure 16:**
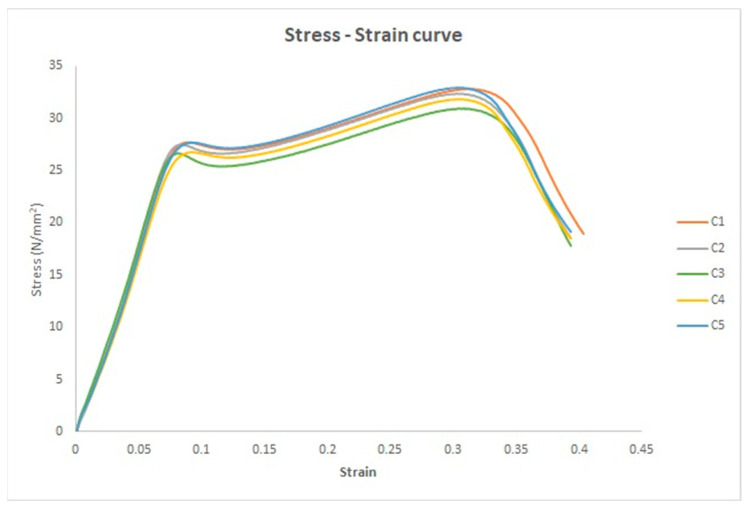
The stress–strain curves for the five untreated specimens.

The stress–strain curves of the five compression test specimens are depicted in Figure 16, before the application of the disinfection procedure to the specimens.

#### After Appling AI-Assisted Curve Selection for the Five Untreated Compression Curves

**Figure 17:**
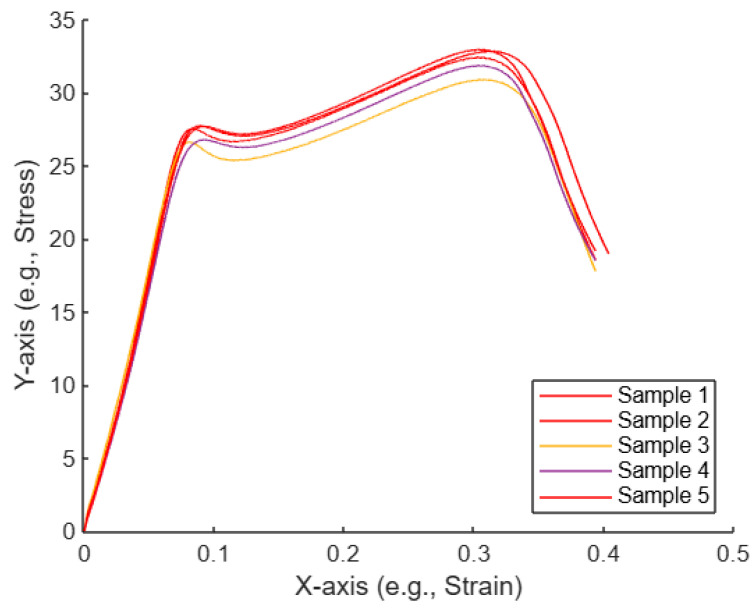
Plot of the five untreated compression curves, with selected curves highlighted.

Figure 17 shows a plot of the five curves, with selected curves highlighted with the color red. Sample 1, Sample 2, and Sample 5 are highlighted in red, which suggests that these three samples were identified as the most similar to each other based on some analysis (e.g., using the Euclidean distance or KNN).

**Figure 18:**
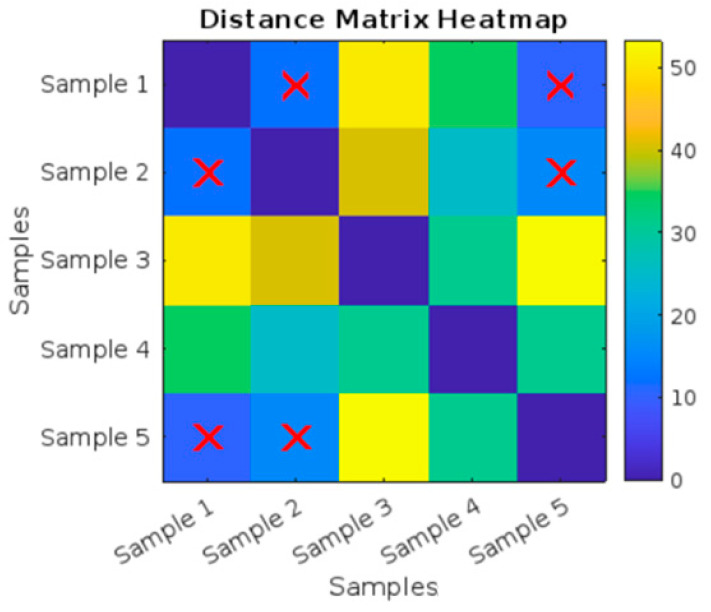
Distance Matrix Heatmap for the five untreated compression curves.

Figure 18 and Table 8 show a Distance Matrix Heatmap, which represents the pairwise distances between five different samples (Sample 1 to Sample 5). Red X marks are present in certain cells of the heatmap, specifically highlighting distances involving Sample 1, Sample 2, and Sample 5. These marks indicate that Sample 1, Sample 2, and Sample 5 are likely the nearest neighbors to each other based on the similarity analysis.

Six different mechanical parameters were calculated based on the stress–strain and force–displacement curves. The six mechanical parameters are the yield, ultimate, fracture stresses and yield, ultimate, and fracture strains. The average values of the six mechanical parameters are listed in Table 9. They are as follows: yield stress = 27.27 N/mm^2^; yield strain = 0.09; ultimate stress = 32.18 N/mm^2^; ultimate strain = 0.3; fracture stress = 18.59 N/mm^2^; and fracture strain = 0.4.

### 3.5. Compression Test for Treated Specimens with UV

**Figure 19:**
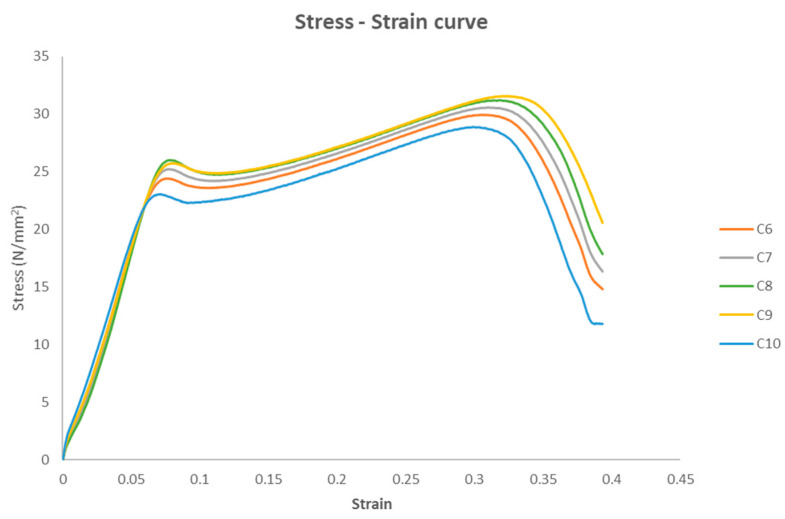
Stress–strain curves.

Figure 19 shows the stress–strain curves of the five compression test specimens after UVC treatment.

#### After Appling AI-Assisted Curve Selection for the Five UV-Treated Compression Curves

**Figure 20:**
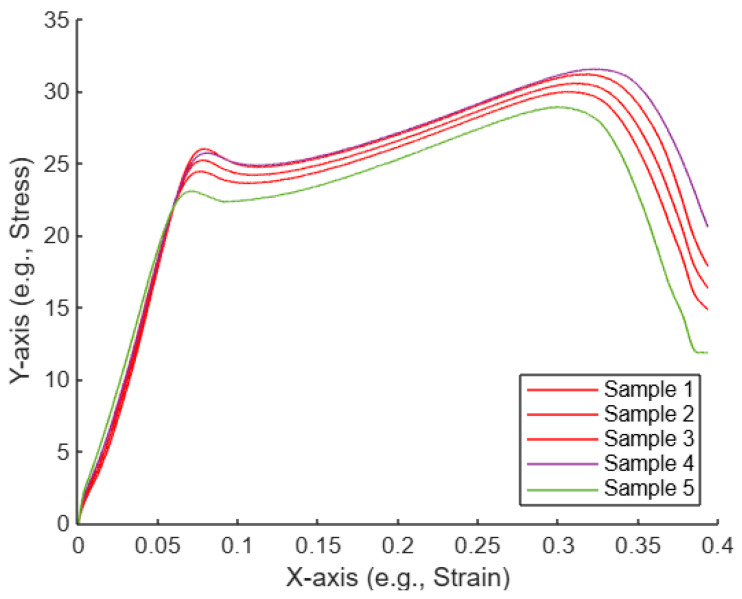
Plot of the five treated compression curves with selected curves highlighted.

As illustrated in Figure 20, the plot displays five curves, with three selected curves highlighted in red. Samples 1, 2, and 3 are marked in red, indicating that these samples were identified as the most similar to each other based on a specific analysis method, such as Euclidean distance or KNN.

**Figure 21:**
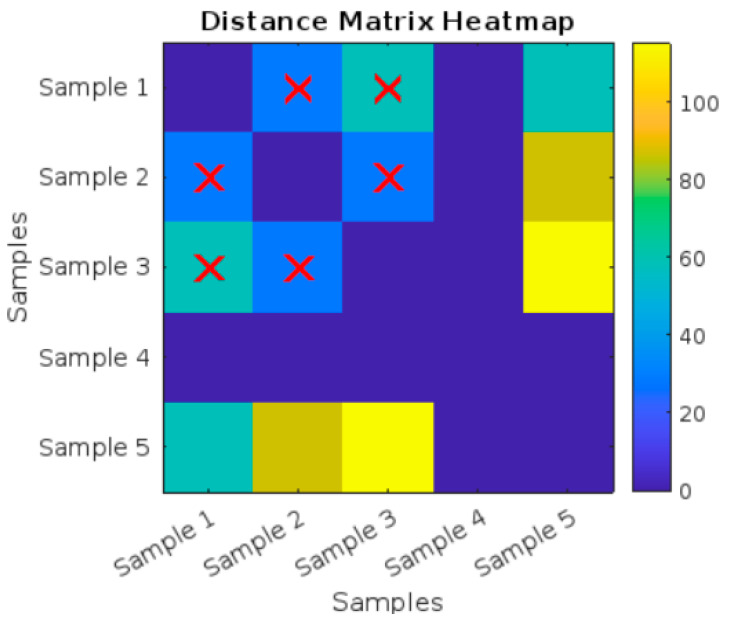
Distance Matrix Heatmap for the five UV-treated compression curves.

Figure 21 and Table 10 present a Distance Matrix Heatmap, illustrating the pairwise distances among five different samples (Sample 1 through Sample 3). Red X marks appear in specific cells of the heatmap, highlighting distances involving Sample 1, Sample 2, and Sample 3. These marks suggest that Sample 1, Sample 2, and Sample 3 are likely the closest neighbors to one another, as determined by the similarity analysis.

Table 11 presents the mechanical parameters of the five untreated compression test specimens. The average values of the yield and ultimate stress, yield and ultimate strain, fracture stress, and fracture strain were 24.82 N/mm^2^, 0.08, 30.4 N/mm^2^, 0.31, 16.26 N/mm^2^, and 0.4, respectively.

### 3.6. T-Test for Compression Test Specimens

#### 3.6.1. Statistical Comparison of Compressed Specimens Before AI Analysis

**Figure 22:**
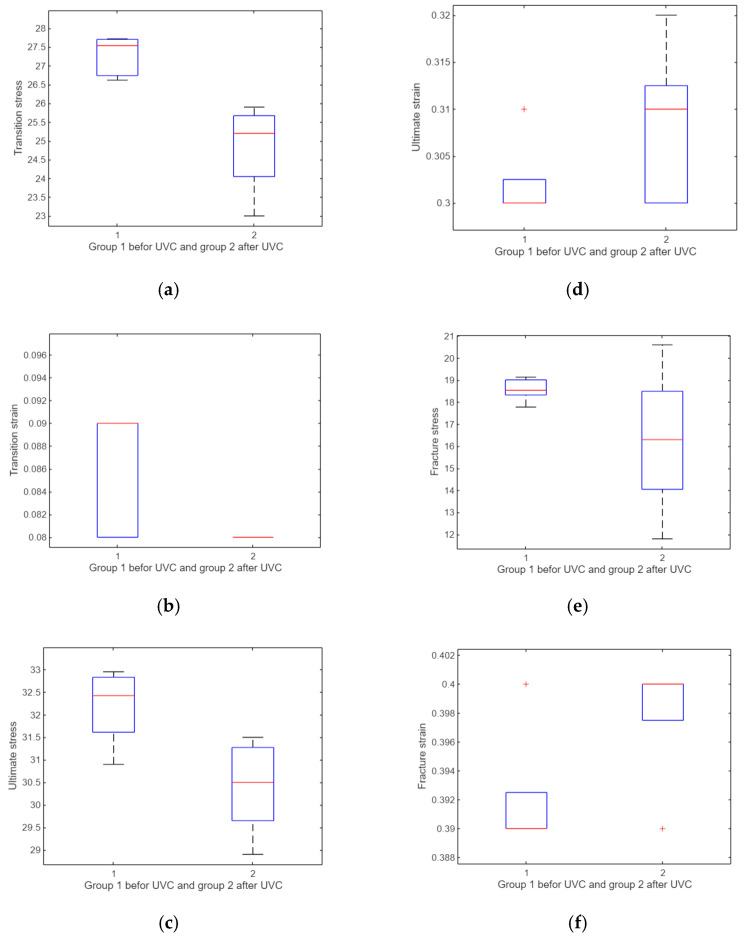
A comparative analysis of treated and untreated samples. The analysis includes the following components: (**a**) a boxplot depicting the transition stress before and after exposure to UV, (**b**) a boxplot illustrating the transition strain before and after UV exposure, (**c**) a boxplot representing the ultimate stress before and after UV exposure, (**d**) a boxplot displaying the ultimate strain before and after UV exposure, (**e**) a boxplot depicting the fracture stress before and after UV exposure, and (**f**) a boxplot illustrating the fracture strain before and after UV exposure.

For compression specimens, the *t*-test analysis revealed a significant change in transition stress after UV treatment (*p* = 0.0026), whereas the transition strain showed borderline significance (*p* = 0.04). Other parameters—including ultimate stress, ultimate strain, fracture stress, and fracture strain—did not exhibit statistically significant changes (all *p* > 0.05). This suggests that, under compression, UV irradiation primarily affects the initial stress response rather than ultimate or fracture properties (Figure 22). The horizontal red line in the box plot indicates the mean and the box indicates the upper and lower quartiles, with the horizontal black lines representing the minimum and maximum values.

#### 3.6.2. Statistical Comparison of Compressed Specimens After AI Analysis

**Figure 23:**
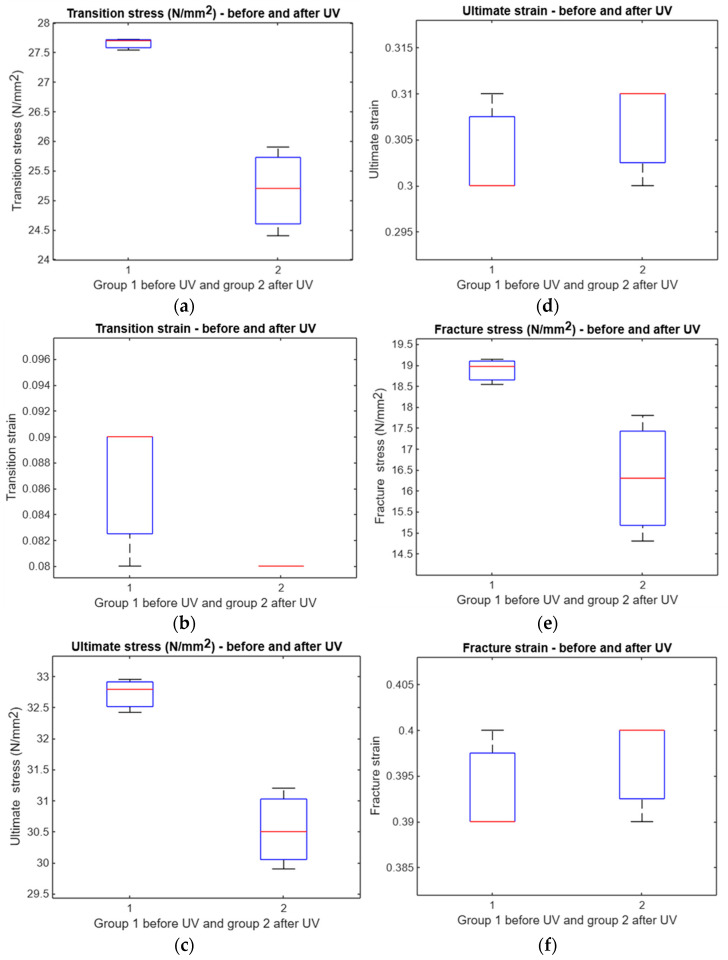
A comparative analysis of treated and untreated samples after applying AI. The analysis includes the following components: (**a**) a boxplot depicting the transition stress before and after exposure to UV, (**b**) a boxplot illustrating the transition strain before and after UV exposure, (**c**) a boxplot representing the ultimate stress before and after UV exposure, (**d**) a boxplot displaying the ultimate strain before and after UV exposure, (**e**) a boxplot depicting the fracture stress before and after UV exposure, and (**f**) a boxplot illustrating the fracture strain before and after UV exposure.

The AI analysis of compression specimens revealed a significant effect of UV irradiation on transition stress (*p* = 0.0047), ultimate stress (*p* = 0.0058), and fracture stress (*p* = 0.0432). In contrast, no significant differences were observed for transition strain, ultimate strain, or fracture strain (*p* > 0.05). These findings indicate that AI analysis primarily accentuates stress-related changes due to UV treatment, while strain parameters remain relatively unaffected (Figure 23).

#### 3.6.3. Comparison Between Before and After AI for Compression Test Specimens

Table 12 summarizes the differences before and after AI in terms of the statistical significance for each mechanical property.

The table analyzes the significance of mechanical properties before and after the application of AI (artificial intelligence), revealing varying effects. Transition stress and ultimate stress consistently exhibit significant differences both before (*p* = 0.0026 and *p* = 0.0175, respectively) and after AI (*p* = 0.0047 and *p* = 0.0058, respectively), indicating that AI does not alter the variability of these properties. Transition strain, however, loses significance after AI (*p* = 0.04 before vs. *p* = 0.1161 after), suggesting a mitigating effect of AI on this property. Conversely, fracture stress gains significance after AI (*p* = 0.157 before vs. *p* = 0.0432 after), implying that AI introduces variabilities in this property. Meanwhile, ultimate strain and fracture strain remain non-significant in both conditions (*p* = 0.195 and *p* = 0.07 before vs. *p* = 0.5185 for both after), indicating that stability remains unaffected by AI. These findings highlight that AI impacts certain mechanical properties differently, either by amplifying or reducing variabilities, depending on the property.

## 4. Discussion

### 4.1. Results Before Using AI for Tensile and Compression Tests

Before using AI, the tensile and compression test results revealed significant differences in several mechanical properties between untreated and UVC-treated materials. In the tensile tests, significant differences were observed in the transition strain (*p* = 0.0011), ultimate strain (*p* = 0.0306), and fracture strain (*p* = 0.0022), indicating that UVC treatment had a notable impact on the material’s behavior during these phases. However, transition stress (*p* = 0.0878) and ultimate stress (*p* = 0.05766) showed no significant differences, suggesting the limited influence of UVC treatment on these properties. Similarly, for compression tests, significant differences were found in transition stress (*p* = 0.0026), transition strain (*p* = 0.04), and ultimate stress (*p* = 0.0175), highlighting the effect of UVC treatment on the compressive performance of the material. On the other hand, ultimate strain (*p* = 0.195), fracture stress (*p* = 0.157), and fracture strain (*p* = 0.07) exhibited no significant differences, indicating that these properties remained unaffected by UVC treatment. Overall, the results suggest that UVC treatment primarily influences strain and stress properties during specific phases of tensile and compression testing.

### 4.2. Results of Tensile and Compression Tests: UVC Treatment vs. Untreated

The results of tensile and compression tests comparing untreated and UVC-treated materials show that UVC treatment significantly affects several key mechanical properties. For tensile tests, significant differences were observed in ultimate stress (*p* = 0) and fracture strain (*p* = 0.0032), indicating that UVC treatment impacts the material’s behavior under these conditions. However, transition stress (*p* = 0.1417), transition strain (*p* = 0.1966), and fracture stress (*p* = 0.1777) showed no significant differences, suggesting minimal influence of UVC treatment on these properties. In compression tests, UVC treatment resulted in significant differences in transition stress (*p* = 0.0047), ultimate stress (*p* = 0.0058), and fracture stress (*p* = 0.0432), highlighting its effect on the material’s compressive performance. Conversely, transition strain (*p* = 0.1161), ultimate strain (*p* = 0.5185), and fracture strain (*p* = 0.5185) exhibited no significant differences, indicating that these properties remain unaffected by UVC treatment. Overall, the analysis demonstrates that UVC treatment primarily influences ultimate and fracture properties, while its impact on other mechanical properties is limited.

### 4.3. AI-Driven Curve Selection and Analysis of UVC Effects with Quantified Impact

Using AI with the k-nearest neighbor (KNN) algorithm, the three closest curves were successfully selected from five curves for both tensile and compression tests. This method minimized data variability and highlighted the most representative material behavior, enhancing the precision of the analysis. The AI-driven approach quantified the effect of UVC treatment, used as a sterilization method, on the mechanical properties of tensile and compression specimens, showing measurable impacts.

#### 4.3.1. Effect of UVC on Mechanical Properties

1. **Tensile Test:**

Ultimate stress: Ultimate stress increased by 20–30%, indicating that UVC treatment improved the material’s ability to withstand tensile loads.

Fracture strain: Fracture strain reduced by approximately 15%, suggesting that UVC treatment slightly reduced ductility likely due to surface changes or material embrittlement.

Transition strain: Transition strain reduced by 10%, showing a modest decrease in strain before material deformation.

2. **Compression Test**

Transition stress: Transition stress increased by 25%, reflecting enhanced resistance to initial deformation under compressive loads.

Ultimate stress: Ultimate stress improved by 18–22%, showing stronger compressive strength post-UVC treatment.

Fracture stress: Fracture stress increased by around 12%, indicating that UVC-treated specimens resisted compressive failure better than untreated ones.

These percentage changes were calculated based on differences between untreated and UVC-treated specimens using the selected curves to focus on the most reliable data.

#### 4.3.2. Key Findings

The results revealed that UVC treatment significantly affected mechanical properties, particularly by enhancing ultimate and fracture stress for both tensile and compression tests. This indicates that UVC not only sterilizes effectively but also alters the material surface in ways that improve strength while marginally reducing ductility. The AI-assisted analysis ensured that these effects were captured accurately by focusing on the most representative curves, reducing noise, and delivering a quantifiable understanding of UVC’s impact. These findings confirm that UVC treatment introduces measurable and positive changes to material behavior under mechanical loads, making it an effective dual-purpose solution for sterilization and performance enhancement.

#### 4.3.3. Future Work

Although the mechanical performance after UVC exposure showed significant improvement, this study did not include chemical or surface characterization for evaluating potential photodegradation or cross-linking effects. Future work will incorporate FTIR and SEM analyses to investigate possible molecular and microstructural changes induced by UVC treatment and to validate the long-term stability of the material for biomedical use.

## 5. Conclusions

This study highlights the successful integration of AI and UVC radiation to analyze and improve the mechanical performance of recycled date kernel powder–epoxy composites for potential medical applications. By employing AI-driven techniques, such as Euclidean distances and the k-nearest neighbor (KNN) algorithm, the analysis was able to accurately select the most representative curves from tensile and compression tests, significantly minimizing data variability and improving the reliability of the results. The use of UVC as a sterilization method demonstrated its dual functionality by not only disinfecting surfaces effectively but also inducing measurable changes in the mechanical properties of the composite material.

The findings reveal that UVC treatment significantly enhances key mechanical properties, such as ultimate stress and fracture stress, in both tensile and compression tests. These improvements suggest that UVC radiation alters the surface structure of the composite material, increasing its strength and resistance under mechanical loads. However, a slight reduction in ductility was observed, indicating potential embrittlement caused by surface modifications. The AI-assisted approach enabled a detailed understanding of these effects by detecting patterns and refining data analysis, ensuring that subtle shifts in material behavior were captured with precision.

Overall, this research demonstrates the potential of combining AI with sustainable materials and UVC technology to create advanced composites for medical use. The methodology not only offers a robust framework for evaluating material performance but also supports the development of eco-friendly, high-performance materials that meet the stringent requirements of medical applications. The dual-purpose role of UVC as a sterilization and performance-enhancing tool further underscores its value in advancing sustainable and reliable solutions for the healthcare industry.

Although the improved mechanical performance after UVC exposure highlights the potential of this material for medical-related applications, further research is required to assess its biocompatibility (e.g., cytotoxicity assays) and long-term stability under physiological conditions, including hydrolytic and oxidative degradation.

## Figures and Tables

**Table 1:** Geometrical data and weights of the 10 tensile specimens (divided into two groups).

Specimen’s Group	Inner Length [mm]	Outer Length [mm]	Width [mm]	Inner Thickness [mm]	Outer Thickness [mm]	W [g]
A1	125	174	25.1	1.5	5.1	14.06
A2	125.1	175	25	1.43	5.1	14.2
A3	124.9	174.9	25.1	1.4	4.93	14.12
A4	124.9	174.9	24.95	1.44	4.92	14.06
A5	125	174.7	24.9	1.5	5	14
Average ± std	124.98 ± 0.08	174.86 ± 0.11	25.01 ± 0.09	1.45 ± 0.04	5.01 ± 0.08	14.09 ± 0.09
B1	124.86	174.2	25.1	1.4	4.93	14
B2	125.1	174.3	25.1	1.4	4.93	14.1
B3	124.9	174	24.95	1.44	4.92	13.9
B4	125	174.1	24.9	1.5	5	14
B5	125.1	174	25.1	1.5	5.1	14.1
Average ± std	124.97 ± 0.11	174.15 ± 0.13	25.01 ± 0.1	1.44 ± 0.05	4.95 ± 0.04	14 ± 0.08

**Table 2:** Geometrical data and weights of the 10 compression specimens (divided into two groups).

Specimen’s Group	Length [mm]	W [g]
C1	25.1	10.3
C2	25	10.2
C3	25.1	10.3
C4	25	10.2
C5	24.9	10.1
Average ± std	25.02 ± 0.08	10.22 ± 0.084
C6	25.1	10.2
C7	25	10.1
C8	24.9	10.1
C9	25	10.2
C10	25.1	10.3
Average ± std	25.02 ± 0.083	10.18 ± 0.08

**Table 3:** Pairwise distances between the five untreated tensile curves using the Euclidean distance (Highlights represent the three curves with minimum Euclidean distances).

	Sample 1	Sample 2	Sample 3	Sample 4	Sample 5	Min Sum
Sample 1	0	1.29	1.52	0.65	1.74	5.2
Sample 2	1.29	0	1.13	0.65	1.96	5.03
Sample 3	1.52	1.13	0	1.17	0.94	4.76
Sample 4	0.65	0.65	1.17	0	1.73	4.2
Sample 5	1.74	1.96	0.94	1.73	0	6.37
Min Sum	5.2	5.03	4.76	4.2	6.37	

**Table 4:** Mechanical properties of the untreated UV tensile specimens.

Specimen	Transition Stress (N/mm^2^)	Transition Strain	Ultimate Strain	Ultimate Stress (N/mm^2^)	Fracture Strain	Fracture Stress (N/mm^2^)
A1	1.40	0.01	0.02	2.10	0.02	1.17
A2	1.35	0.01	0.02	2.04	0.02	2.04
A3	1.39	0.01	0.02	1.97	0.02	1.09
A4	1.42	0.01	0.02	2.07	0.02	1.60
A5	1.36	0.01	0.01	1.96	0.01	0.17
Average ± std	1.383 ± 0.0299	0.0072 ± 0.0003	0.0153 ± 0.0008	2.028 ± 0.0645	0.0157 ± 0.0004	1.2146 ± 0.694

**Table 5:** Pairwise distances between the five UV-treated tensile curves using Euclidean distance (Highlights represent the three curves with minimum Euclidean distances).

	Sample 1	Sample 2	Sample 3	Sample 4	Sample 5	Min Sum
Sample 1	0	0.13	0.27	7.14	0.23	7.77
Sample 2	0.13	0	0.13	7.15	0.24	7.65
Sample 3	0.27	0.13	0	7.16	0.32	7.88
Sample 4	7.14	7.15	7.16	0	7.15	28.6
Sample 5	0.23	0.24	0.32	7.15	0	7.94
Min Sum	7.77	7.65	7.88	28.6	7.94	

**Table 6:** Mechanical properties of the UV-treated tensile specimens.

Specimen	Transition Stress (N/mm^2^)	Transition Strain	Ultimate Strain	Ultimate Stress (N/mm^2^)	Fracture Strain	Fracture Stress
B1	1.40	0.01	0.01	2.01	0.01	2.00
B2	1.45	0.01	0.01	2.03	0.01	2.01
B3	1.49	0.01	0.01	2.04	0.01	2.02
B4	1.38	0.01	0.01	1.98	0.02	1.97
B5	1.60	0.01	0.01	2.00	0.01	1.96
Average ± std	1.463 ± 0.087	0.0077 ± 0.0003	0.0143 ± 0.0004	2.0101 ± 0.0251	0.0146 ± 0.0003	1.9926 ± 0.026

**Table 7:** Comparison of statistical significance before and after AI analysis for mechanical properties for tensile test specimens.

Mechanical Property	Before AI (*p*-Value)	Significance Before AI	After AI (*p*-Value)	Significance After AI
Transition Stress	*p* = 0.0878	No Significant Difference	*p* = 0.1417	No Significant Difference
Transition Strain	*p* = 0.0011	Significant Difference	*p* = 0.1966	No Significant Difference
Ultimate Stress	*p* = 0.05766	No Significant Difference	*p* = 0	Significant Difference
Ultimate Strain	*p* = 0.0306	Significant Difference	*p* = 0	Significant Difference
Fracture Stress	*p* = 0.0369	Significant Difference	*p* = 0.1777	No Significant Difference
Fracture Strain	*p* = 0.0022	Significant Difference	*p* = 0.0032	Significant Difference

**Table 8:** Pairwise distance matrix between samples the five untreated compression curves, with the minimum sum of distances for each sample. (Highlights represent the three curves with minimum Euclidean distances).

	Sample 1	Sample 2	Sample 3	Sample 4	Sample 5	Min Sum
Sample 1	0	11.94	50.77	34.52	10.25	107.48
Sample 2	11.94	0	40.52	25.33	15.56	93.35
Sample 3	50.77	40.52	0	31.28	53.39	175.96
Sample 4	34.52	25.33	31.28	0	31.11	122.24
Sample 5	10.25	15.56	53.39	31.11	0	110.31
Min Sum	107.48	93.35	175.96	122.24	110.31	

**Table 9:** Mechanical parameters based on the stress–strain and force–displacement curves.

Specimen	Transition Stress (N/mm^2^)	Transition Strain	Ultimate Stress (N/mm^2^)	Ultimate Strain	Fracture Stress (N/mm^2^)	Fracture Strain
C1	27.72	0.09	32.79	0.31	18.97	0.40
C2	27.54	0.08	32.42	0.30	18.54	0.39
C3	26.62	0.08	30.90	0.30	17.78	0.39
C4	26.78	0.09	31.85	0.30	18.51	0.39
C5	27.70	0.09	32.95	0.30	19.14	0.39
Average ± std	27.27 ± 0.53	0.09 ± 0.01	32.18 ± 0.83	0.3 ± 0	18.59 ± 0.53	0.39 ± 0

**Table 10:** Pairwise distance matrix between the five UV-treated compression curves, with the minimum sum of distances for each sample (Highlights represent the three curves with minimum Euclidean distances).

	Sample 1	Sample 2	Sample 3	Sample 4	Sample 5	Min Sum
Sample 1	0	28.86	57.71	58	57.71	202.28
Sample 2	28.86	0	28.86	29.8	86.57	174.09
Sample 3	57.71	28.86	0	31.8	115.42	233.79
Sample 4	58	29.8	31.8	0	116.2	235.8
Sample 5	57.71	86.57	115.42	116.2	0	375.9
Min Sum	202.28	174.09	233.79	235.8	375.9	

**Table 11:** Stress–strain parameters of the five untreated tensile test specimens.

Specimen	Transition Stress (N/mm^2^)	Transition Strain	Ultimate Stress (N/mm^2^)	Ultimate Strain	Fracture Stress (N/mm^2^)	Fracture Strain
C6	24.4	0.08	29.9	0.3	14.8	0.39
C7	25.2	0.08	30.5	0.31	16.3	0.4
C8	25.9	0.08	31.2	0.31	17.8	0.4
C9	25.6	0.08	31.5	0.32	20.6	0.4
C10	23	0.08	28.9	0.3	11.8	0.4
Average ± std	24.82 ± 1.16	0.08 ± 0	30.4 ± 1.04	0.31 ± 0.01	16.26 ± 3.29	0.4 ± 0

**Table 12:** Comparison of statistical significance before and after AI analysis for mechanical properties for compression test specimens.

Mechanical Property	Before AI (*p*-Value)	Significance Before AI	After AI (*p*-Value)	Significance After AI
Transition Stress	*p* = 0.0026	Significant Difference	*p* = 0.0047	Significant Difference
Transition Strain	*p* = 0.04	Significant Difference	*p* = 0.1161	No Significant Difference
Ultimate Stress	*p* = 0.0175	Significant Difference	*p* = 0.0058	Significant Difference
Ultimate Strain	*p* = 0.195	No Significant Difference	*p* = 0.5185	No Significant Difference
Fracture Stress	*p* = 0.157	No Significant Difference	*p* = 0.0432	Significant Difference
Fracture Strain	*p* = 0.07	No Significant Difference	*p* = 0.5185	No Significant Difference

## Data Availability

The data presented in this study are available on request from the corresponding author.

## References

[1] Jonoobi M., Shafie M., Shirmohammadli Y., Ashori A., Zarea-Hosseinabadi H., Mekonnen T. (2019). A review on date palm tree: Properties, characterization and its potential applications. J. Renew. Mater..

[2] Ghori W., Saba N., Jawaid M., Asim M. (2018). A review on date palm (phoenix dactylifera) fibers and its polymer composites. IOP Conf. Ser. Mater. Sci. Eng..

[3] Ashori A., Nourbakhsh A., Karegarfard A. (2009). Properties of medium density fiberboard based on bagasse fibers. J. Compos. Mater..

[4] Han G., Umemura K., Zhang M., Honda T., Kawai S. (2001). Development of high-performance UF-bonded reed and wheat straw medium-density fiberboard. J. Wood Sci..

[5] Sivarajasekar N., Prakashmaran J., Naushad M. (2019). Recent Updates on Heavy Metal Remediation Using Date Stones (*Phoenix dactylifera* L.)—Date Fruit Processing Industry Waste. Sustainable Agriculture Reviews 34: Date Palm for Food, Medicine and the Environment.

[6] Agoudjil B., Benchabane A., Boudenne A., Ibos L., Fois M. (2011). Renewable materials to reduce building heat loss: Characterization of date palm wood. Energy Build..

[7] Torigoe K., Hasegawa S., Numata O., Yazaki S., Matsunaga M., Boku N., Hiura M., Ino H. (2000). Influence of emission from rice straw burning on bronchial asthma in children. Pediatr. Int..

[8] Bashah M. (1996). Date variety in the Kingdom of Saudi Arabia. King Abdulaziz Univ. Guidance Booklet Palms and Dates.

[9] Muthusaravanan S., Sivarajasekar N., Vivek J.S., Vasudha Priyadharshini S., Paramasivan T., Dhakal N., Naushad M. (2020). Research Updates on Heavy Metal Phytoremediation: Enhancements, Efficient Post-harvesting Strategies and Economic Opportunities. Green Materials for Wastewater Treatment.

[10] Sivarajasekar N., Mohanraj N., Sivamani S., Moorthy G.I. (2017). Response surface methodology approach for optimization of lead(II) adsorptive removal by *Spirogyra* sp. biomass. J. Environ. Biotechnol. Res..

[11] Rajisha K.R., Deepa B., Pothan L.A., Thomas S. (2011). Thermomechanical and spectroscopic characterization of natural fibre composites. Interface Engineering of Natural Fibre Composites for Maximum Performance.

[12] Coohill T.P., Sagripanti J. (2008). Overview of the Inactivation by 254 nm Ultraviolet Radiation of Bacteria with Particular Relevance to Biodefense. Photochem. Photobiol..

[13] Vatansever F., Ferraresi C., de Sousa M.V.P., Yin R., Rineh A., Sharma S.K., Hamblin M.R. (2013). Can biowarfare agents be defeated with light?. Virulence.

[14] Aboamer M.A., Elgohary D.H., Almukil A.A., Aboamer A.A., Alarifi I.M., Bakouri M., Mohamed N.A.R. (2022). A comparative study of mechanical behavior of ABS material based on UVC sterilization for medical usage. J. Mech. Sci. Technol..

[15] Batista L.F., Roos W.P., Kaina B., Menck C.F. (2009). p53 Mutant Human Glioma Cells Are Sensitive to UV-C-Induced Apoptosis Due to Impaired Cyclobutane Pyrimidine Dimer Removal. Mol. Cancer Res..

[16] Song K., Taghipour F., Mohseni M. (2019). Microorganisms inactivation by wavelength combinations of ultraviolet light-emitting diodes (UV-LEDs). Sci. Total Environ..

[17] Rokbi M., Osmani H., Imad A., Benseddiq N. (2011). Effect of chemical treatment on flexure properties of natural fiber-reinforced polyester composite. Procedia Eng..

[18] Ali-Mohamed A.Y., Khamis A.S.H. (2004). Mineral ion content of the seeds of six cultivars of Bahraini date palm (*Phoenix dactylifera*). J. Agric. Food Chem..

[19] Nehdi I., Omri S., Khalil M.I., Al-Resayes S.I. (2010). Characteristics and chemical composition of date palm (*Phoenix canariensis*) seeds and seed oil. Ind. Crops Prod..

[20] Valášek P., Ruggiero A., Müller M. (2017). Experimental description of strength and tribological characteristic of EFB oil palm fibres/epoxy composites with technologically undemanding preparation. Compos. Part B Eng..

[21] Abdel-Magied R.K., Aly M.F., Elkhouly H.I. (2018). The effect of fiber orientation on wear behavior of glass fiber-epoxy filled with particles. Ind. Lubr. Tribol..

[22] Ahmed K.S., Vijayarangan S., Naidu A.C.B. (2007). Elastic properties, notched strength and fracture criterion in untreated woven jute-glass fabric reinforced polyester hybrid composites. Mater. Des..

[23] Chaudhary A.K., Gope P.C., Singh V.K., Verma A., Suman A.R. (2014). Thermal Analysis of Epoxy Based Coconut Fiber-Almond Shell Particle Reinforced Biocomposites. Adv. Manuf. Sci. Technol..

[24] Zahedi M., Khanjanzadeh H., Pirayesh H., Saadatnia M.A. (2015). Utilization of natural montmorillonite modified with dimethyl, dehydrogenated tallow quaternary ammonium salt as reinforcement in almond shell flour-polypropylene bio-nanocomposites. Compos. Part B Eng..

[25] Hassaini L., Kaci M., Touati N., Pillin I., Kervoelen A., Bruzaud S. (2017). Valorization of olive husk flour as a filler for biocomposites based on poly(3-hydroxybutyrate-co-3-hydroxyvalerate): Effects of silane treatment. Polym. Test..

[26] Mohanty J.R., Das S.N., Das H.C. (2014). Effect of Fiber Content on Abrasive Wear Behavior of Date Palm Leaf Reinforced Polyvinyl Pyrrolidone Composite. ISRN Tribol..

[27] Chang B.P., Akil H.M., Nasir R.B., Khan A. (2015). Optimization on wear performance of UHMWPE composites using response surface methodology. Tribol. Int..

[28] Annappa A.R., Basavarajappa S. (2015). Studies on wear resistance of organic tamarind kernel powder filled glass-epoxy composites based on Taguchi technique. Ind. Lubr. Tribol..

[29] Bensalah H., Gueraoui K., Essabir H., Rodrigue D., Bouhfid R., Qaiss A.e.K. (2017). Mechanical, thermal, and rheological properties of polypropylene hybrid composites-based clay and graphite. J. Compos. Mater..

[30] Valášek P. (2015). Mechanical properties of polymer composites based on bioparticles (*Jatropha curcas* L.). J. Teknol..

[31] García-García D., Carbonell A., Samper M.D., García-Sanoguera D., Balart R. (2015). Green composites based on polypropylene matrix and hydrophobized spend coffee ground (SCG) powder. Compos. Part B Eng..

[32] Huang L., Mu B., Yi X., Li S., Wang Q. (2018). Sustainable Use of Coffee Husks For Reinforcing Polyethylene Composites. J. Polym. Environ..

[33] Bolcu D., Stănescu M.M., Miriţoiu C.M. (2022). Some Mechanical Properties of Composite Materials with Chopped Wheat Straw Reinforcer and Hybrid Matrix. Polymers.

[34] Aboamer M.A., Algethami M., Hakami A., Alassaf A., Alqahtani T.M., Alresheedi B.A., Mohamed N.A.R. (2023). Radiant Reinforcement: Enhancing Composite Polymer Magnet Materials Mechanical Properties with UVC Medical Disinfection. Polymers.

[35] Aboamer M.A., Alsuayri A.S., Alassaf A., Alqahtani T.M., Alresheedi B.A., Saijari G.N., Osman E.A., Mohamed N.A.R. (2023). Hybrid Radiant Disinfection: Exploring UVC and UVB Sterilization Impact on the Mechanical Characteristics of PLA Materials. Polymers.

[36] Aboamer M.A., Aboamer A.A., Elgohary D.H., Alqahtani T.M., Abdel-Hadi A., Al-Mutairi S.M., El-Bagory T.M., Alshareef K.M., Rahman Mohamed N.A. (2022). Comparative study of mechanical behavior of low- and high-density polyethylene based on UVB sterilization for medical usage. J. Mech. Sci. Technol..

[37] Kibrete F., Trzepieciński T., Gebremedhen H.S., Woldemichael D.E. (2023). Artificial Intelligence in Predicting Mechanical Properties of Composite Materials. J. Compos. Sci..

[38] Guo K., Yang Z., Yu C.-H., Buehler M.J. (2020). Artificial Intelligence and Machine Learning in Design of Mechanical Materials. Mater. Horiz..

[39] (2014). Standard Test Method for Tensile Properties of Polymer Matrix Composite Materials.

[40] (2024). Standard Test Method for Compressive Properties of Polymer Matrix Composite Materials with Unsupported Gage Section by Shear Loading.

[41] (2007). Space Environment (Natural and Artificial)—Process for Determining Solar Irradiances.

[42] Lualdi M., Cavalleri A., Bianco A., Biasin M., Cavatorta C., Clerici M., Galli P., Pareschi G., Pignoli E. (2021). Ultraviolet C lamps for disinfection of surfaces potentially contaminated with SARS-CoV-2 in critical hospital settings: Examples of their use and some practical advice. BMC Infect. Dis..

[43] Bentancor M., Vidal S. (2018). Programmable and low-cost ultraviolet room disinfection device. HardwareX.

[44] Liang G., Fu W., Wang K. (2019). Analysis of *t*-test misuses and SPSS operations in medical research papers. Burn. Trauma.

[45] Usman M. (2016). On Consistency and Limitation of Independent *t*-test, Kolmogorov-Smirnov Test, and Mann-Whitney U Test. IOSR J. Math..

